# The effect of haptoglobin genotype on the association of asymmetric dimethylarginine and *DDAH 1* polymorphism with diabetic macroangiopathy

**DOI:** 10.1186/s12933-022-01702-6

**Published:** 2022-12-02

**Authors:** Shiyun Wang, Zixuan Deng, Hong Zhang, Rong Zhang, Dandan Yan, Xiaojiao Zheng, Weiping Jia, Cheng Hu

**Affiliations:** 1 Department of Endocrinology and Metabolism, Shanghai Sixth People’s Hospital Affiliated to Shanghai Jiao Tong University School of Medicine, Shanghai Diabetes Institute, Shanghai Key Laboratory of Diabetes Mellitus, Shanghai Clinical Center for Diabetes, 600 Yishan Road, Shanghai, 200233 People’s Republic of China; 2 Center for Translational Medicine, Shanghai Diabetes Institute, Department of Endocrinology and Metabolism, Shanghai Sixth People’s Hospital Affiliated to Shanghai Jiao Tong University School of Medicine, 600 Yishan Road, Shanghai, 200233 People’s Republic of China; 3 Institute for Metabolic Disease, Fengxian Central Hospital Affiliated to Southern Medical University, 6600 Nanfeng Road, 201499 Shanghai, People’s Republic of China

**Keywords:** Haptoglobin, Dimethylarginine dimethylaminohydrolase, Asymmetric dimethylarginine, Single nucleotide polymorphism, Diabetic macroangiopathy

## Abstract

**Background:**

Dimethylarginine dimethylaminohydrolase (DDAH) 1 maintains the bioavailability of nitric oxide by degrading asymmetric dimethylarginine (ADMA). Here, we aimed to investigate the effect of haptoglobin (Hp) genotype on the association of ADMA and *DDAH 1* polymorphism with diabetic macroangiopathy.

**Methods:**

In stage 1, 90 Chinese participants with type 2 diabetes were enrolled to measure a panel of targeted metabolites, including ADMA, using tandem mass spectrometry (BIOCRATES AbsoluteIDQ™ p180 kit). In stage 2, an independent cohort of 2965 Chinese patients with type 2 diabetes was recruited to analyze the effect of Hp genotype on the association between *DDAH 1* rs233109 and diabetic macroangiopathy. Hp genotypes were detected using a validated assay based on the TaqMan method. *DDAH 1* rs233109 was genotyped by matrix-assisted laser desorption/ionization time-of-flight mass spectroscopy using the MassARRAY platform.

**Results:**

In stage 1, serum ADMA levels correlated with common Hp genotypes (β ± SE = − 0.049 ± 0.023, *P* = 0.035), but not with diabetic macroangiopathy (*P* = 0.316). In stage 2, the distribution of *DDAH 1* rs233109 genotype frequencies was 15% (CC), 47% (TC), and 38% (TT), which was in Hardy-Weinberg equilibrium (*P* = 0.948). A significant Hp genotype by rs 233109 genotype interaction effect on diabetic macroangiopathy was found (*P* = 0.017). After adjusting for confounders, patients homozygous for rs233109 CC were more likely to develop diabetic macroangiopathy than those carrying TT homozygotes in the Hp 2-2 subgroup [odds ratio = 1.750 (95% confidence interval, 1.101–2.783), *P* = 0.018].

**Conclusion:**

Hp genotype affects the association between *DDAH 1* rs233109 and diabetic macroangiopathy in Chinese patients with type 2 diabetes.

**Supplementary Information:**

The online version contains supplementary material available at 10.1186/s12933-022-01702-6.

## Background

According to the International Diabetes Federation [1], 537 million adults live with diabetes worldwide, and an estimated 240 million people have undiagnosed diabetes. Type 2 diabetes is the most common type of diabetes, accounting for over 90% of all diabetes cases and leading to several disabling and life-threatening complications, such as cardiovascular and cerebrovascular diseases [2]. Although the underlying etiology remains uncertain, inflammation and dysfunction of the endothelium play a key role in the development of diabetic macroangiopathy [3–5]. The endothelium is essential for maintaining vascular homeostasis, ensuring a balance between vasoactive factors, such as angiotensin type II and nitric oxide (NO), which control its permeability, adhesiveness, and integrity. However, this balance is impaired when diabetes occurs [6].

Asymmetric dimethylarginine (ADMA), an endogenous inhibitor of nitric oxide synthase (NOS) [7], is considered to be a risk factor for the development of endothelial dysfunction and cardiovascular diseases [8]. A recent meta-analysis based on 11 studies with 9496 participants reported that elevated blood ADMA concentration was an independent predictor of all-cause and cardiovascular mortality in patients with coronary artery disease [9]. There are two isoforms of dimethylarginine dimethylaminohydrolase (DDAH) that metabolize ADMA. Among these, DDAH 1 is the predominant isoform that maintains NO bioavailability by degrading ADMA [10]. Valkonen et al. [11] hypothesized that several functional variations in genes coding DDAH enzymes in different populations exist. They reported the finding of a *DDAH 1* gene mutation affecting blood levels of ADMA and incidence of cardiovascular diseases.

As a natural antioxidant in the circulatory system, haptoglobin (Hp) is an acute-phase protein that binds free hemoglobin (Hb) to prevent heme-mediated oxidative damage [12]. The Hb–Hp complex is rapidly cleared from the bloodstream by binding to the CD163 scavenger receptor on the surface of macrophages [13]. The Hp gene is characterized by a genetic polymorphism with two different alleles (Hp 1 and Hp 2), resulting in three common genotypes: Hp 1-1, Hp 2-1, and Hp 2-2. Several differences have been reported between the different Hp genotypes with regard to antioxidative properties, CD163 binding functions, and angiogenesis effects [14]. The role of Hp polymorphisms and their association with diabetic complications have been extensively studied [15]. We previously determined that Chinese patients with type 2 diabetes carrying the Hp 1 allele exhibit a higher level of oxidative stress and an increased susceptibility to diabetic macroangiopathy [16].

Growing evidence exists that oxidative stress impairs the activity of DDAH, leading to increased ADMA concentrations in diabetes [17, 18]. This highlights the importance of ADMA as a possible risk factor and indicates the potential role of Hp genotypes that may influence the association between *DDAH 1* polymorphism and susceptibility to diabetic macrovascular complications. In this study, we measured a panel of metabolites, including ADMA, using a targeted metabolomics approach and explored whether Hp genotypes influence the association of ADMA and single nucleotide polymorphism (SNP) of the *DDAH 1* gene with diabetic macroangiopathy in Chinese patients with type 2 diabetes.

## Methods

### Study population

The present study was conducted in compliance with the Declaration of Helsinki and approved by the Institutional Review Board of Shanghai Sixth People’s Hospital. Each patient provided written informed consent for participation. In stage 1, we studied 90 unrelated Chinese patients with type 2 diabetes (30 for each of the three common Hp genotypes: Hp 1-1, Hp 2-1, and Hp 2-2), enrolled from April 2012 to February 2014, whose details were included in the Shanghai Diabetes Institute inpatient database. In stage 2, we studied another independent cohort of 2965 unrelated Chinese patients with type 2 diabetes enrolled between January 2003 and February 2005 in the Shanghai Diabetes Institute inpatient database. Diabetes was defined according to the 1999 World Health Organization criteria, with no change in hypoglycemic regimen in the past three months. Participants who presented severe diabetic infection or diabetes-related emergencies (diabetic ketoacidosis, hyperglycemic hyperosmolar state, and severe hypoglycemic events), severe renal or hepatic dysfunction, psychiatric disorders, cancer, pregnancy or lactation, hemolytic disease, or drug or alcohol addiction in the 3 months were excluded from this study.

### Anthropometric and clinical measurements

Height (m) and weight (kg) were measured and body mass index (BMI) was calculated as “weight (kg)/height squared (m^2^)”. Systolic and diastolic blood pressures (mm Hg) were measured three times by an experienced physician using a mercury sphygmomanometer and then averaged. Blood samples from all patients were obtained after 8–10 h of overnight fasting immediately after admission. Serum samples were collected via centrifugation and stored at − 80 °C until assayed. Serum concentrations of triglyceride (TG), total cholesterol (TC), high-density lipoprotein cholesterol (HDL-C), and low-density lipoprotein cholesterol (LDL-C) were measured using a Hitachi 7600-020 automated Analyser (Hitachi, Tokyo, Japan). Hemoglobin A1c (HbA1c) levels were determined via high-performance liquid chromatography using a Bio-Rad Variant II hemoglobin testing system (Bio-Rad Laboratories, Hercules, CA, USA).

### Metabolite measurements

A targeted metabolomics approach was applied to detect the concentrations of a panel of metabolites in the blood using an AbsoluteIDQ™ p180 Kit (BIOCRATES Life Sciences AG, Innsbruck, Austria). A total of 184 metabolites, including 21 amino acids, 21 biogenic amines, 1 hexose, 40 acylcarnitines, 87 glycerophospholipids [14 lyso-phosphatidylcholines (lysoPCs) and 73 phosphatidylcholines (PCs)], and 14 sphingolipids (SMs) were measured using this kit. The assay procedures followed the manufacturer’s instructions, and the metabolite nomenclature has been previously described in detail [19]. Amino acids and biogenic amines were quantified by liquid chromatography-tandem mass spectrometry using internal standards, whereas flow injection analysis-tandem mass spectrometry was applied for acylcarnitines, glycerophospholipids, SMs, and hexoses. A Waters XEVO™ TQ mass spectrometer coupled with a Waters ACQUITY ultra-performance liquid chromatograph (Waters, Manchester, UK) and Biocrates MetIQ™ software (BIOCRATES Life Sciences AG, Innsbruck, Austria) were used to automatically measure and quantify metabolite concentrations. The metabolite concentrations were reported in µmol/L (µM) and exported to an Excel file. Metabolites with missing values > 20% and/or measured values below the detection limit or above the highest calibration criteria were excluded, resulting in 168 metabolites for further analysis.

### Genotyping of polymorphisms

Genomic DNA was extracted from peripheral blood leukocytes in blood samples. The Hp genotypes were determined using TaqMan assays on the 7900HT Fast Real-Time Polymerase Chain Reaction System (Applied Biosystems, Foster City, CA, USA), with modifications to a previously described method [16, 20]. SNP rs233109 of *DDAH 1* was selected for genotyping by primer extension of multiplex products with detection by matrix-assisted laser desorption/ionization time-of-flight mass spectrometry (MALDI-TOF MS) using the Sequenom MassARRAY platform (MassARRAY Compact Analyzer; Sequenom, San Diego, CA, USA).

### Diagnosis of diabetic macroangiopathy

Diabetic macroangiopathy was defined in patients with type 2 diabetes who met any of the following criteria: (a) diagnosis of carotid atherosclerosis and/or lower limb arteriosclerosis based on the presence of carotid and/or lower limb arterial plaque (a focal protrusion ≥ 50% of the surrounding intima-media or intima-media thickness ≥ 1.5 mm or arterial lumen encroaching > 0.5 mm) or significant stenosis (stenosis ≥ 50%) measured by vascular color Doppler ultrasound, as previously reported [21]; (b) diagnosis of cerebral hemorrhage and/or stroke based on neurological signs and symptoms in combination with brain computed tomography and/or magnetic resonance imaging; and (c) diagnosis of cardiovascular diseases based on a history of myocardial infarction (clinical ischemic symptoms, electrocardiographic and biochemical markers of myocardial necrosis changes) and/or coronary angioplasty.

### Statistical analyses

The data were analyzed using SAS for Windows (version 9.2; SAS Institute, Cary, NC, USA). Differences in basic traits among patients with different genotypes were tested using the chi-squared test for categorical variables. For continuous variables with normal or skewed distributions, ANOVA or Kruskal-Wallis tests were conducted. Correlations between common Hp genotypes and blood metabolites were analyzed using multiple linear regression with adjustment for confounding factors.

The Hardy-Weinberg equilibrium calculator (http://www.oege.org/software/hwe-mr-calc.shtml) was used to test the Hardy-Weinberg equilibrium for each variant [22]. Linkage disequilibrium between the common Hp genotype and rs233109 genotype, including |D′| and r^2^, was estimated using PLINK (version 1.07; http://pngu.mgh.harvard.edu/~purcell/plink/) [23]. In stage 2, considering the low frequency of the Hp1-1 genotype (< 10%), the Hp 1-1 and Hp 2-1 genotypes were combined into a subgroup of Hp 1 carriers for further analysis. The association between rs233109 and diabetic macroangiopathy was analyzed under an additive model using multivariable logistic regression analysis, and odds ratios (ORs) with 95% confidence intervals (CIs) have been presented. Furthermore, the effect of the interaction between Hp genotypes and rs233109 genotypes on the occurrence of diabetic macroangiopathy was tested under different models.

Based on an estimated effect size of genetic loci for diabetic macroangiopathy (~ 1.25), our samples had > 80% power to detect an SNP with a minor allele frequency of 0.35 at a level of significance of 0.05.

## Results

### Serum ADMA levels in stage 1

**Fig. 1:**
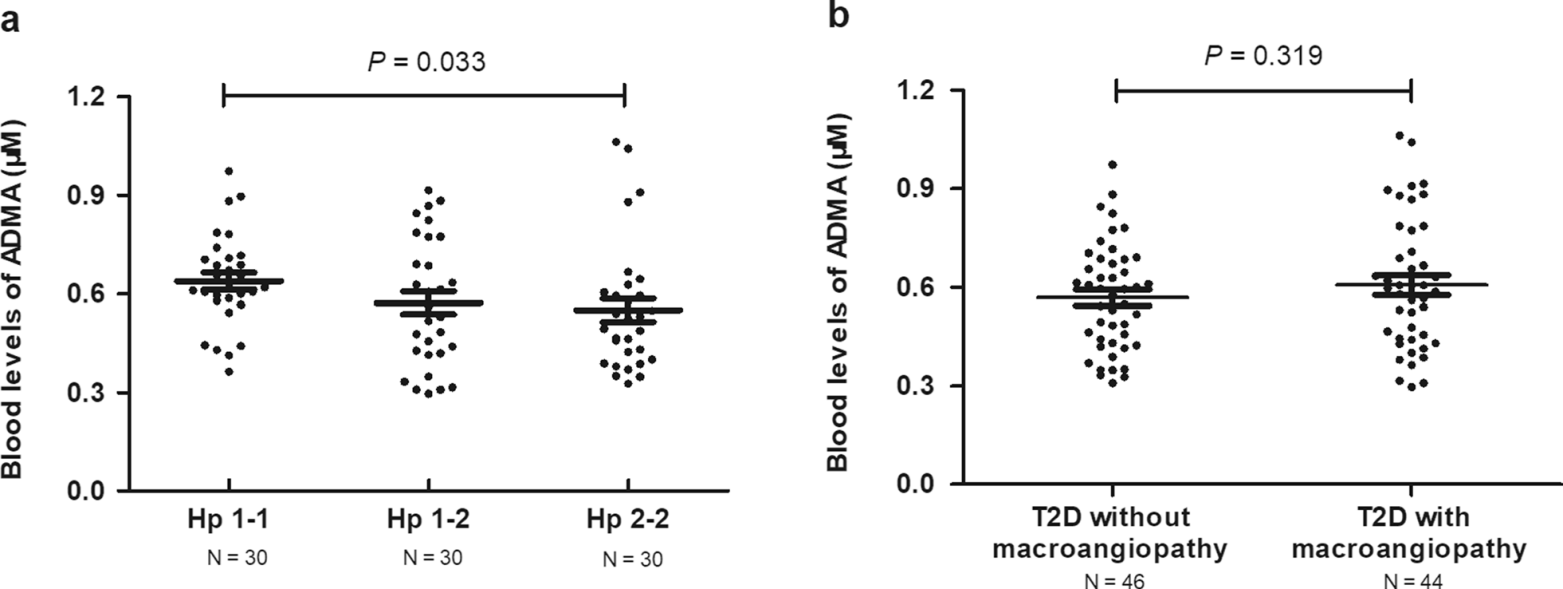
Blood ADMA levels grouped by common Hp genotype and diabetic macroangiopathy **a** Blood ADMA levels were lower in patients carrying the Hp 2 allele (*P* = 0.033). **b** No difference in blood ADMA levels between patients with and without diabetic macroangiopathy (*P* = 0.319). ADMA, asymmetric dimethylarginine; Hp, haptoglobin; T2D, type 2 diabetes. Blood ADMA levels are shown as dot plots; the mean is indicated by the middle black solid line. The standard error of the mean is indicated by the bottom and top black solid lines

The basic clinical characteristics in stage 1 are shown in Table 1. A total of 23 blood metabolites were found to be significantly different among the different common Hp genotypes (*P* < 0.05, Additional file 1: Table S1). Among them, serum ADMA levels were higher in patients carrying the Hp 1 allele (*P* = 0.033, Fig. 1a). After adjusting for age, sex, BMI, blood pressure, smoking, duration of diabetes, HbA1c, TC, and TG, serum ADMA levels remained significantly and independently correlated with common Hp genotypes (β ± SE = − 0.049 ± 0.023, *P* = 0.035). Serum ADMA levels between patients with and without diabetic macroangiopathy were shown in Fig. 1b (*P* = 0.319) and no association was found between ADMA and diabetic macroangiopathy (*P* = 0.316).

**Table 1:** Basic clinical characteristics of 90 participants

Variable	Hp 1-1	Hp 2-1	Hp 2-2	*P* value
N	30	30	30	–
Age (years)	57.1 ± 10.7	56.6 ± 14.0	57.0 ± 11.5	0.978
Male/female (n)	24/6	18/12	19/11	0.170
BMI (kg/m^2^)	25.0 ± 2.8	24.2 ± 2.8	25.2 ± 4.6	0.722
SBP (mmHg)	130 (120, 150)	130 (120, 150)	130 (120, 135)	0.281
DBP (mmHg)	80 (70, 86)	80 (70, 90)	80 (70, 85)	0.996
Duration of diabetes (years)	10.5 (7.0, 15.0)	10.0 (4.0, 14.0)	10.0 (6.0, 15.0)	0.721
HbA1c (%)	8.1 (7.1, 10.1)	8.3 (7.6, 10.9)	8.6 (6.9, 9.6)	0.629
HbA1c [mmol/mol]	65 (54, 87)	67 (60, 96)	70 (52, 81)	
Total cholesterol (mmol/L)	4.4 ± 0.9	4.6 ± 0.9	4.6 ± 1.0	0.497
Triglycerides (mmol/L)	1.4 ± 0.9	1.6 ± 0.9	1.7 ± 1.0	0.104
HDL-C (mmol/L)	1.0 ± 0.3	1.1 ± 0.3	1.0 ± 0.2	0.886
LDL-C (mmol/L)	2.6 ± 0.8	2.7 ± 0.8	2.8 ± 0.7	0.778
Diabetic macroangiopathy	17 (56.67%)	14 (46.67%)	13 (43.33%)	0.304

Data are shown as n (%), mean ± standard deviation, or median (interquartile range).

*Hp* haptoglobin, *Hp 1 carriers* Hp 1–1 and Hp 2 − 1, *BMI* body mass index, *SBP* systolic blood pressure, *DBP* diastolic blood pressure, *HbA1c* hemoglobin A1c, *HDL-C* high-density lipoprotein cholesterol, *LDL-C* low-density lipoprotein cholesterol

### Basic clinical characteristics in stage 2

The distribution of rs233109 genotype frequencies was 15% (CC, n = 438), 47% (TC, n = 1401), and 38% (TT, n = 1126), which were in Hardy-Weinberg equilibrium (*P* = 0.948). The clinical characteristics of the 2965 patients grouped by the rs233109 genotype are presented in Additional file 1: Table S2. There was no difference in the prevalence of diabetic macroangiopathy among individuals with different rs233109 genotypes (*P* = 0.117).

Common Hp genotypes were distributed as follows: Hp 1-1, 8% (n = 233); Hp 2-1, 38% (n = 1138); and Hp 2-2, 54% (n = 1594). These genotypes were in Hardy-Weinberg equilibrium (*P* = 0.135). Additionally, SNP rs233109 was in ultralow linkage disequilibrium with common Hp genotypes (|D′| = 0.015, r^2^ < 0.001). Considering the low frequency of the Hp1-1 genotype (< 10%), the Hp 1-1 and Hp 2 -1 genotypes were combined into a subgroup of Hp 1 carriers for further analyses [24, 25].

**Fig. 2:**
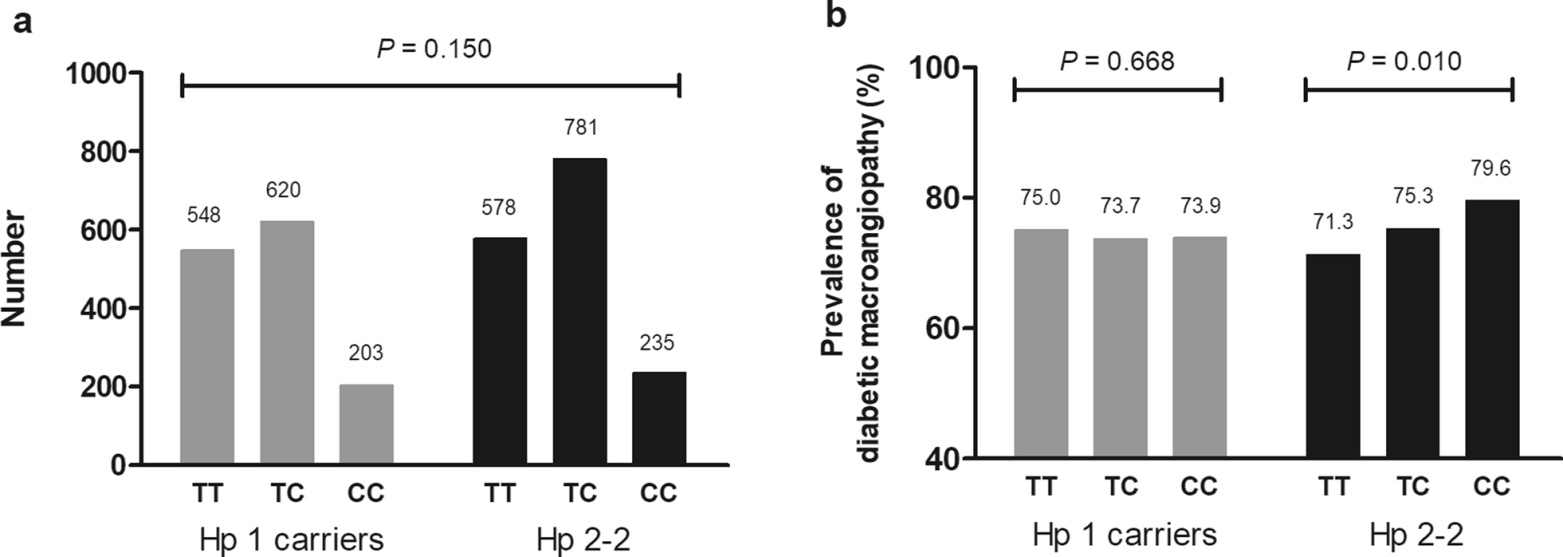
Genotypic distribution of rs233109 and prevalence of diabetic macroangiopathy grouped by Hp genotype **a** No difference in the genotypic distribution of rs233109 between the two Hp subgroups (*P* = 0.150). **b** A significant difference in the prevalence of diabetic macroangiopathy in participants with Hp 2-2 (*P* = 0.010) but not in Hp 1 carriers (*P* = 0.668). Hp, haptoglobin; Hp 1 carriers, Hp 1-1 and Hp 2-1. TT, TC, and CC are rs233109 genotypes. The Hp 1 carriers and Hp 2-2 subgroup are shown as gray bars and black bars, respectively

The clinical characteristics of the 2965 patients grouped by the two Hp subgroups are shown in Table 2. The TC and LDL-C levels differed between the two subgroups (*P* < 0.001). The genotypic distributions of rs233109 grouped into the two Hp subgroups are shown in Fig. 2a, and no difference was found in rs233109 genotypic distribution between the two Hp subgroups (*P* = 0.150). The prevalence of diabetic macroangiopathy grouped by rs233109 genotype between the two Hp subgroups is shown in Fig. 2b, and a higher prevalence of diabetic macroangiopathy was found in individuals who were CC homozygotes of rs233109 than in the T allele carriers in the Hp 2-2 subgroup (*P* = 0.010).

**Table 2:** Basic clinical characteristics of 2965 participants

Variable	Hp 1 Carriers	Hp 2-2	*P* value
Number	1371	1594	–
Age (years)	61.3 ± 11.8	61.9 ± 11.9	0.126
Male/female (n)	727 / 644	803 / 791	0.150
Body mass index (kg/m^2^)	24.6 ± 6.1	24.5 ± 3.6	0.834
Systolic blood pressure (mmHg)	130 (120, 145)	130 (120, 150)	0.718
Diastolic blood pressure (mmHg)	80 (74, 90)	80 (75, 90)	0.097
Duration of diabetes (years)	7.0 (2.0, 12.0)	7.0 (2.0, 12.0)	0.840
HbA1c (%)	9.1 ± 2.2	9.0 ± 2.2	0.376
HbA1c [mmol/mol]	75.0 ± 24.0	74.0 ± 24.0	
Total cholesterol (mmol/L)	4.6 (3.9, 5.3)	4.7 (4.2, 5.5)	< 0.001
Triglycerides (mmol/L)	1.4 (1.0, 2.1)	1.5 (1.0, 2.1)	0.115
HDL-C (mmol/L)	1.1 (0.9, 1.3)	1.1 (0.9, 1.3)	0.087
LDL-C (mmol/L)	2.9 (2.4, 3.5)	3.0 (2.5, 3.6)	< 0.001
Diabetic macroangiopathy disease	1018 (74.3%)	1187 (74.5%)	0.894

Data are shown as n (%), mean ± standard deviation, or median (interquartile range)

*Hp* haptoglobin, *Hp 1 carriers* Hp 1-1 and Hp 2-1, *HbA1c* hemoglobin A1c, *HDL-C* high-density lipoprotein cholesterol, *LDL-C* low-density lipoprotein cholesterol

### Interaction between Hp genotypes and SNP rs233109 on diabetic macroangiopathy

After adjusting for age, sex, BMI, duration of diabetes, HbA1c, blood pressure, TC, and LDL-C, no association was observed between rs233109 and diabetic macroangiopathy [OR = 1.119 (95% CI, 0.959–1.306), *P* = 0.153]. A significant Hp genotype by rs 233109 genotype interaction effect on diabetic macroangiopathy was found (*P* = 0.017, Table 3). In subgroup analyses, we found that patients who were CC homozygotes for rs233109 were more likely to develop diabetic macroangiopathy than those carrying TT homozygotes in the Hp 2-2 subgroup [OR = 1.750 (95% CI, 1.101–2.783), *P* = 0.018] (Table 3). In Hp 1 carriers, no significant association was found between the *DDAH 1* polymorphism rs233109 and diabetic macroangiopathy (*P* > 0.05, Table 3).

**Table 3:** Association of rs233109 with diabetic macroangiopathy grouped by Hp genotype

	Hp 1 Carriers			Hp 2–2	
	OR (95% CI)	*P* value		OR (95% CI)	*P* value
Crude					
CC	0.943 (0.653, 1.363)	0.756		1.570 (1.090, 2.261)	0.015
TC	0.935 (0.718, 1.216)	0.614		1.228 (0.963, 1.565)	0.098
TT	1 (Reference)			1 (Reference)	
*P* for interaction		0.037			
Model 1					
CC	0.846 (0.531, 1.348)	0.481		1.626 (1.038, 2.547)	0.034
TC	0.917 (0.660, 1.276)	0.609		1.370 (1.012, 1.856)	0.042
TT	1 (Reference)			1 (Reference)	
*P* for interaction		0.021			
Model 2					
CC	0.846 (0.527, 1.359)	0.490		1.750 (1.101, 2.783)	0.018
TC	0.944 (0.673, 1.323)	0.737		1.386 (1.015, 1.892)	0.040
TT	1 (Reference)			1 (Reference)	
*P* for interaction		0.017			

*DDAH1* rs233109 genotypes: CC, TC, and TT. Model 1 was adjusted for age, sex, BMI, duration of diabetes, and HbA1c. Model 2 included variables in model 1 plus blood pressure, TC, and LDL-C

*Hp* haptoglobin; *Hp 1 carriers* Hp 1-1 and Hp 2-1

## Discussion

The current study explored the effect of Hp genotype on the association of ADMA and *DDAH 1* gene polymorphisms with diabetic macroangiopathy. In stage 1, we found that serum ADMA level was correlated with common Hp genotypes. In stage 2, a significant Hp genotype by *DDAH 1* rs233109 interaction effect on diabetic macroangiopathy was found. Furthermore, individuals with CC homozygotes of the rs233109 polymorphism in *DDAH 1* were more likely to develop diabetic macroangiopathy than those carrying TT homozygotes in the Hp 2-2 subgroup. In Hp 1 carriers, no significant association was found between the *DDAH 1* polymorphism rs233109 and diabetic macroangiopathy.

As a novel risk factor, the relationship between ADMA and cardiovascular disease has been extensively studied in basic and clinical research [26]. In the presence of high levels of ADMA, NOS is uncoupled to produce peroxynitrite, which results in decreased NO bioavailability and increased oxidative stress [27]. Moreover, DDAH activity was inhibited in hyperglycemia. In diabetic rats, DDAH activity in smooth muscle cells and the endothelium was significantly reduced and negatively correlated with ADMA levels [28]. In human endothelial cells, co-incubation with the antioxidant reversed the effects of the high-glucose conditions on DDAH activity and serum ADMA accumulation [28]. This suggests that oxidative stress is closely related to DDAH activity, which further affects ADMA concentrations in patients with diabetes. Accordingly, differences in genetically endowed antioxidant properties may confer increased or decreased ADMA levels and different susceptibilities to the development of diabetic macroangiopathy.

Hp serves as an antioxidant, owing to its ability to prevent Hb-induced oxidative tissue and vascular damage. Due to the existence of genetic polymorphisms in Hp, there are functional differences in the antioxidant capacity, CD163 binding functions, and angiogenesis effects of different Hp proteins [29]. The Hp genotype is a major determinant of CVD risk in patients with diabetes. Several studies evaluating the susceptibility gene for cardiovascular disease in diabetes have shown that patients with the Hp 2-2 genotype have as much as a 5-fold increased risk of cardiovascular disease compared to those with the Hp 1-1 genotype [15]. Results from our previous study combined with findings by Gurung et al. [30] demonstrated that Chinese patients with type 2 diabetes who carried the Hp 2-2 genotype had a lower prevalence of diabetic macroangiopathy.

In the current study, ADMA levels were correlated with common Hp genotypes and were lowest in patients with the Hp 2-2 genotype. This may be due to the stronger antioxidant capacity of the Hp 2-2 genotype, which partially offsets the inhibitory effect of diabetes on DDAH enzyme activity, leading to a lower level of ADMA. Further research should be conducted to confirm the underlying mechanism. Additionally, we failed to find a significant association between ADMA levels and diabetic macroangiopathy. However, we conducted a subgroup analysis and found a difference in serum ADMA levels between patients with and without diabetic macroangiopathy in the Hp 2-2 subgroup (*P* = 0.036, Additional file 1: Figure S1). Moreover, a significant association was identified between serum ADMA levels and diabetic macroangiopathy in the Hp 2-2 subgroup (*P* = 0.043). However, after adjusting for age, sex, BMI, blood pressure, smoking, duration of diabetes, HbA1c, TC, and TG levels, this correlation became non-significant (*P* = 0.161), most likely due to small sample size. Cohort studies with larger sample sizes are needed to explore this relationship further [31].

There are two isoforms of DDAH, namely DDAH 1 and DDAH 2. DDAH 1 is mainly found in the proximal tubules of the kidney and liver, which are organs that extract ADMA from circulation. DDAH 1 has been reported to protect mice against high-fat diet-induced hepatic steatosis and insulin resistance [32]. DDAH 2 is the predominant isoform in the vasculature, including endothelial cells, intracellular vesicles, and vascular smooth muscle cells. Experiments in vivo showed that *Ddah 1* −/− and *Ddah* +/− mice had increased circulating ADMA levels, whereas silencing of *Ddah 2* attenuated endothelium-dependent vasodilation [33, 34]. The apparent rate of ADMA metabolism of DDAH 1 was estimated to be 70 times higher than that of DDAH 2, suggesting that DDAH 1 may regulate NOS activity and endothelial function through an ADMA-dependent mechanism [35]. Therefore, modulating the activity and expression of DDAH may be a potential therapeutic approach to influence the circulating levels of ADMA and the progression of metabolic diseases.

Several genetic variants of *DDAH 1* have been shown to be associated with ADMA levels and metabolic diseases [33, 36]. A study of 1016 70-year-old participants showed that the SNPs rs233109, rs12140935, and rs6669293 in *DDAH 1* were strongly associated with ADMA levels, even after adjustment for major cardiovascular risk factors and diseases [37]. Lu et al. [38] found that another SNP, rs1241321, in *DDAH 1* was associated with type 2 diabetes and its long-term adverse cardiovascular events. In this study, we explored the association between *DDAH 1* rs233109 and diabetic macroangiopathy in an independent cohort of 2965 Chinese patients with type 2 diabetes and found that participants with CC homozygotes were more likely to develop diabetic macroangiopathy than those with TT homozygotes in the Hp 2-2 subgroup. We speculate that the primary mechanism by which *DDAH 1* variant rs233109 promotes macrovascular disease may be by regulating circulating concentrations of ADMA to affect endothelial cell function, which is fully exerted in the Hp 2-2 genotypes with higher antioxidant properties. Thus, different clinical strategies for diabetes management can be employed to minimize the risk of diabetic macroangiopathy in patients with varying genetic backgrounds.

To the best of our knowledge, we reported the influence of Hp genotype on the association of *DDAH 1* polymorphism with diabetic macroangiopathy for the first time. The main strengths of this study include its two-stage study design with a large sample size, well-documented clinical characteristics, and the measurement of a panel of targeted metabolites, which enhances the reliability of our results.

This study has several limitations. First, we did not evaluate endothelial dysfunction markers other than ADMA in stage 1. The association between Hp genotype and endothelial dysfunction markers could be further explored in future studies. Second, the serum levels of ADMA were not measured in stage 2 because serum samples were not available. Although rs233109 was strongly associated with ADMA level in Swedes [36], it is unclear whether rs233109 promotes diabetic macroangiopathy by regulating circulating ADMA concentrations in this study. Third, we did not adjust for drug usage and lifestyle, including antioxidative drug usage, alcohol consumption, and smoking status, as confounding factors in this study. This should be considered in future studies to understand potential interactions. Finally, this was a cross-sectional observational study based on hospitalized Chinese patients with type 2 diabetes and may not be applicable to all patients with type 2 diabetes from other ethnic groups. A prospective cohort study is required to provide more robust evidence.

## Conclusion

In summary, we examined the influence of the Hp genotype on the association of *DDAH 1* variant rs233109 with diabetic macroangiopathy in a large Chinese type 2 diabetes cohort. Our data suggest that *DDAH 1* polymorphism may play a crucial role in susceptibility to diabetic macroangiopathy in patients carrying Hp 2-2.

## Supplementary Information

**Additional file 1: Table S1**. Blood metabolites grouped by the Hp genotype. **Table S2**. Basic clinical characteristics of 2965 participants grouped by rs233109 genotype.** Figure S1**. Blood ADMA levels grouped by diabetic macroangiopathy in different Hp genotypes.

## Data Availability

The datasets used and/or analyzed during the current study are available from the corresponding author on reasonable request.

## Abbreviations

ADMA: Asymmetric dimethylarginine
BMI: Body mass index
CIs: Confidence intervals
DDAH: Dimethylarginine dimethylaminohydrolase
Hb: Hemoglobin
HbA1c: Hemoglobin A1c
HDL-C: High-density lipoprotein cholesterol
Hp: Haptoglobin
LDL-C: Low-density lipoprotein cholesterol
MALDI-TOF MS: Matrix-assisted laser desorption/ionization time-of-flight mass spectrometry
NO: Nitric oxide
NOS: Nitric oxide synthase
ORs: Odds ratios
PCs: Phosphatidylcholines
SMs: Sphingolipids
SNP: Single nucleotide polymorphism
TG: Triglyceride
