# Supplementary material for: The effect of haptoglobin genotype on the association of asymmetric dimethylarginine and DDAH 1 polymorphism with diabetic macroangiopathy
Source: Cardiovasc Diabetol. 2022 Dec 2;21:265. doi: 10.1186/s12933-022-01702-6 (PMC9716717; doi:10.1186/s12933-022-01702-6)
Supplement: Supplementary file 1 — Additional file 1: Table S1. Blood metabolites grouped by the Hp genotype. Table S2. Basic clinical characteristics of 2965 participants grouped by rs233109 genotype. Figure S1. Blood ADMA levels grouped by diabetic macroangiopathy in different Hp genotypes. [file 12933_2022_1702_MOESM1_ESM.docx]

**Additional file 1**

**Table S1 Blood metabolites grouped by the Hp genotypes**

| **Metabolites** | **Hp 1-1** | **Hp 2-1** | **Hp 2-2** | ***P* Value** |
| --- | --- | --- | --- | --- |
| Acylcarnitines (*μM*) |  |  |  |  |
| C12:1 | 0.112 ± 0.027 | 0.137 ± 0.042 | 0.151 ± 0.042 | 0.001 |
| C14:1 | 0.200 ± 0.048 | 0.210 ± 0.062 | 0.244 ± 0.064 | 0.021 |
| C14:2 | 0.035 ± 0.035 | 0.049 ± 0.031 | 0.068 ± 0.054 | 0.027 |
| C14:2-OH | 0.006 ± 0.005 | 0.009 ± 0.007 | 0.010 ± 0.007 | 0.008 |
| C16 | 0.102 ± 0.049 | 0.086 ± 0.056 | 0.126 ± 0.067 | 0.043 |
| Lyso-phosphatidylcholines (*μM*) | |  |  |  |
| lysoPC a C26:1 | 0.195 ± 0.114 | 0.239 ± 0.125 | 0.274 ± 0.107 | 0.037 |
| Phosphatidylcholines (*μM*) | |  |  |  |
| PC aa C28:1 | 1.124 ± 0.236 | 1.362 ± 0.455 | 1.300 ± 0.312 | 0.026 |
| PC aa C32:1 | 3.832 ± 1.247 | 4.594 ± 2.071 | 5.303 ± 2.374 | 0.019 |
| PC aa C34:1 | 84.494 ± 22.046 | 97.266 ± 24.664 | 105.722 ± 27.967 | 0.002 |
| PC aa C34:2 | 272.716 ± 55.800 | 311.890 ± 71.462 | 316.007 ± 84.650 | 0.049 |
| PC aa C34:3 | 6.115 ± 1.705 | 7.000 ± 1.692 | 7.444 ± 2.555 | 0.047 |
| PC aa C36:1 | 19.237 ± 6.444 | 20.679 ± 4.482 | 22.243 ± 4.410 | 0.006 |
| PC aa C36:3 | 56.723 ± 14.655 | 63.566 ± 12.938 | 68.317 ± 16.333 | 0.014 |
| PC aa C38:3 | 19.744 ± 7.067 | 21.344 ± 6.276 | 24.462 ± 7.156 | 0.033 |
| PC aa C40:6 | 15.995 ± 6.478 | 16.405 ± 5.808 | 18.851 ± 4.840 | 0.011 |
| Amino Acids (*μM*) |  |  |  |  |
| Asn | 57.744 ± 8.368 | 52.243 ± 7.384 | 56.142 ± 8.544 | 0.018 |
| His | 105.985 ± 12.388 | 105.959 ± 13.731 | 117.740 ± 16.712 | 0.006 |
| Met | 28.084 ± 3.973 | 28.854 ± 6.823 | 31.790 ± 7.067 | 0.044 |
| Phe | 108.609 ± 17.594 | 112.718 ± 20.384 | 120.234 ± 16.696 | 0.024 |
| Trp | 57.349 ± 10.019 | 57.090 ± 12.616 | 65.789 ± 13.626 | 0.018 |
| Tyr | 78.559 ± 15.085 | 79.990 ± 19.782 | 91.939 ± 18.431 | 0.006 |
| Biogenic Amines (*μM*) |  |  |  |  |
| ADMA | 0.639 ± 0.142 | 0.572 ± 0.196 | 0.550 ± 0.195 | 0.033 |
| Serotonin | 0.561 ± 0.233 | 0.452 ± 0.239 | 0.420 ± 0.203 | 0.027 |

Hp: haptoglobin; lysoPC a, lysophosphatidylcholine acyl; PC aa, phosphatidylcholine diacyl; Asn, asparagine; His, histidine; Met, methionine; Phe, phenylalanine; Trp, tryptophan; Tyr, tyrosine; ADMA, asymmetric dimethylarginine.

**Table S2 Basic clinical characteristics of 2965 participants**

**grouped by rs233109 genotype**

| **Variable** | **TT** | **TC** | **CC** | ***P* value** |
| --- | --- | --- | --- | --- |
| N | 1126 | 1401 | 438 | **--** |
| Age (years) | 61.4 ± 11.7 | 61.8 ± 12.1 | 61.9 ± 11.4 | 0.483 |
| Male/female (n) | 610/516 | 705/696 | 215/223 | 0.033 |
| BMI (kg/m^2^) | 24.6 ± 6.5 | 24.5 ± 3.7 | 24.5 ± 3.5 | 0.852 |
| SBP (mmHg) | 130 (120, 145) | 130 (120, 145) | 135 (120, 145) | 0.811 |
| DBP (mmHg) | 80 (75, 90) | 80 (75, 90) | 80 (75, 90) | 0.768 |
| Duration of diabetes (years) | 7.0 (2.0, 11.9) | 7.0 (2.0, 12.0) | 7.0 (2.0, 12.0) | 0.860 |
| HbA1c (%) | 9.0 ± 2.2 | 9.0 ± 2.3 | 9.2 ± 2.2 | 0.311 |
| HbA1c [mmol/mol] | 74.0 ± 24.0 | 74.0 ± 25.0 | 74.0 ± 24.0 |  |
| Total cholesterol (mmol/L) | 4.7 (4.0, 5.4) | 4.7 (4.1, 5.4) | 4.7 (4.1, 5.4) | 0.720 |
| Triglycerides (mmol/L) | 1.4 (1.0, 2.1) | 1.5 (1.0, 2.2) | 1.4 (1.0, 2.1) | 0.170 |
| HDL-C (mmol/L) | 1.1 (0.9, 1.3) | 1.1 (0.9, 1.3) | 1.1 (0.9, 1.3) | 0.819 |
| LDL-C (mmol/L) | 2.9 (2.4, 3.5) | 3.0 (2.4, 3.5) | 3.0 (2.4, 3.6) | 0.716 |
| Diabetic macroangiopathy | 823 (73.1%) | 1045 (74.6%) | 337 (76.9%) | 0.117 |

Data are shown as n (%), mean ± standard deviation, or median (interquartile range). rs233109 genotypes: TT, TC, and CC; BMI, body mass index; SBP, systolic blood pressure; DBP, diastolic blood pressure; HbA1c, hemoglobin A1c; HDL-C: high-density lipoprotein cholesterol; LDL-C: low-density lipoprotein cholesterol.


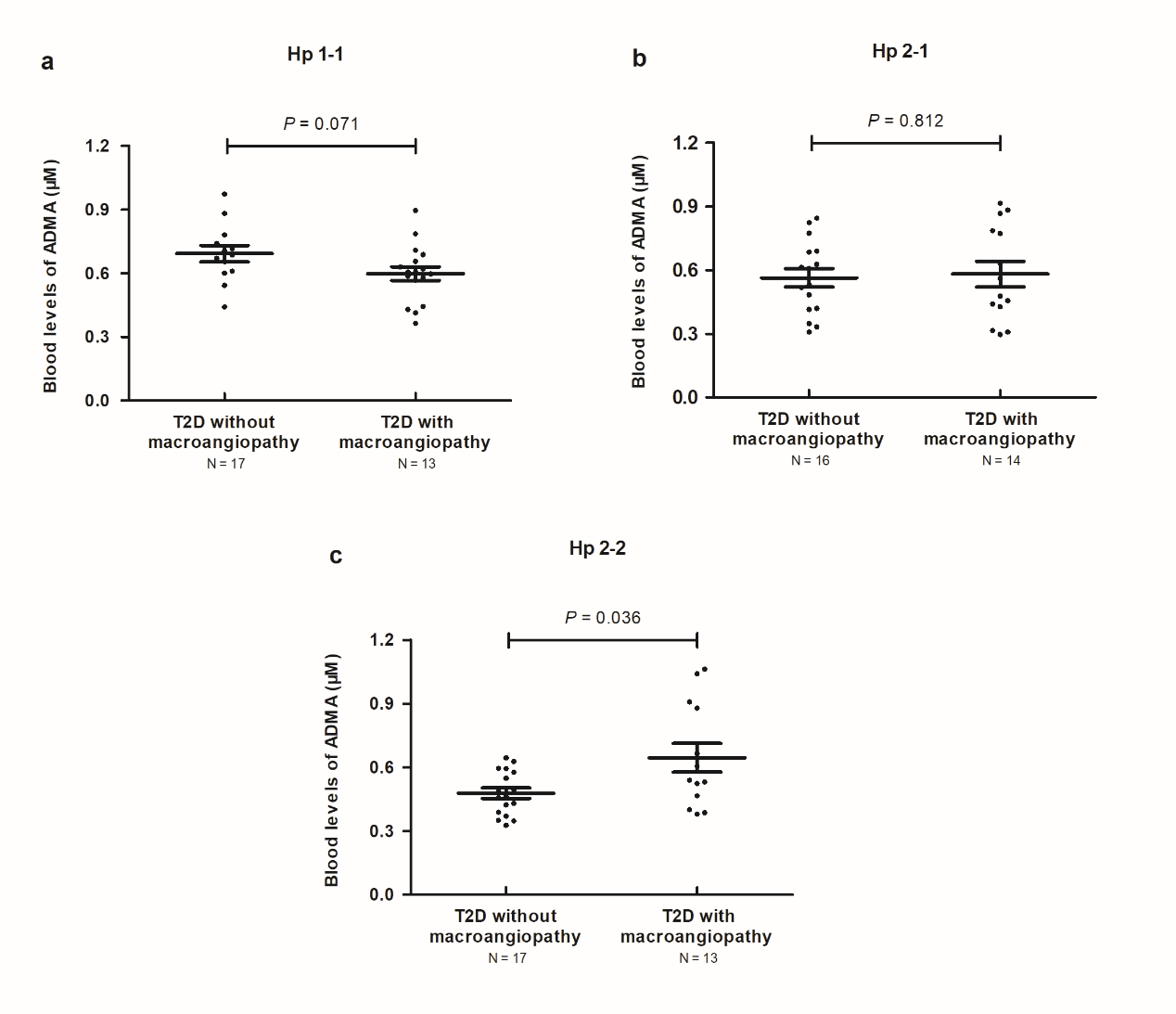


**Figure S1. Blood ADMA levels grouped by diabetic macroangiopathy in different Hp genotypes.**

**a** A trend of differences in blood ADMA levels between patients with and without diabetic macroangiopathy in the Hp 1-1 subgroup (*P* = 0.071). **b** No difference in blood ADMA levels between patients with and without diabetic macroangiopathy in the Hp 2-1 subgroup (*P* = 0.812). **c** A difference in blood ADMA levels between patients with and without diabetic macroangiopathy in the Hp 2-2 subgroup (*P* = 0.036). ADMA, asymmetric dimethylarginine; Hp, haptoglobin; T2D, type 2 diabetes. Blood ADMA levels are shown as dot plots; the mean is indicated by the middle black solid line. The standard error of the mean is indicated by the bottom and top black solid lines.
